# ANCA-Associated Vasculitis after Moderna COVID-19 Vaccination

**DOI:** 10.1155/2023/4906876

**Published:** 2023-04-17

**Authors:** Shiko Gen, Takanori Iwai, Sayuri Ohnari, Kanako Nobe, Naofumi Ikeda

**Affiliations:** Department of Nephrology, Saitama Sekishinkai Hospital, Sayama, Japan

## Abstract

We experienced a case of myeloperoxidase antineutrophil cytoplasmic antibody (MPO-ANCA)-associated vasculitis after Moderna COVID-19 vaccination. An 82-year-old woman developed pyrexia and general malaise one month after her third booster vaccine, and the symptoms persisted. Blood testing revealed inflammation, a high level of MPO-ANCA, and microscopic hematuria. MPO-ANCA-associated vasculitis was diagnosed by renal biopsy. The symptoms improved with steroid therapy. Common adverse reactions to mRNA vaccines against COVID-19 include pyrexia and general malaise, but MPO-ANCA-associated vasculitis can also occur. If pyrexia, prolonged general malaise, urinary occult blood, or renal impairment is observed, the onset of MPO-ANCA-associated vasculitis should be considered.

## 1. Introduction

Infection with severe acute respiratory syndrome coronavirus 2 (SARS-CoV-2) in humans was first recognized in December 2019, and a pandemic of coronavirus disease 2019 (COVID-19) due to SARS-CoV-2 has since occurred throughout the world. The use of recently developed mRNA vaccines, such as BNT162b2 (Pfizer) and mRNA-1273 (Moderna), has provided effective protection against COVID-19 [1, 2]. Common adverse events include mild-to-moderate tenderness at the injection site, fever, fatigue, body aches, and headache [1, 2], but the long-term sequelae of the vaccines remain unknown.

Antineutrophil cytoplasmic antibody (ANCA)-associated vasculitis (AAV) is a small vessel vasculitis hallmarked by the presence of antibodies against autoantigens in cytoplasmic granules of neutrophils [3]. While many case reports have described a temporal association between influenza vaccination and the new onset of AAV [4, 5], few reports have described the development of AAV after COVID-19 vaccination [6, 7]. Here, we report a case of new-onset renal-limited antimyeloperoxidase (MPO)AAV after Moderna COVID-19 vaccination.

## 2. Case Report

An 82-year-old woman with high blood pressure with no history of abnormal urinalysis or renal dysfunction received two vaccinations with the mRNA BNT162b2 COVID-19 vaccine (Pfizer) against the novel coronavirus in May and June, 2021. She received the mRNA-1273 COVID-19 vaccine (Moderna) as a third booster in early February, 2022. The patient developed a body temperature of 37.5°C and headache on the day after vaccination, which resolved in 3 days. However, from early March, she developed a body temperature of 37.5°C–37.9°C and general malaise. She visited the previous doctor 7 days before admission to our hospital. There were no findings suggestive of obvious bacterial infection, but the blood test revealed an inflammatory reaction with high C-reactive protein of 15 mg/dL and white blood cell count of 13000/*μ*L.

**Figure 1:**
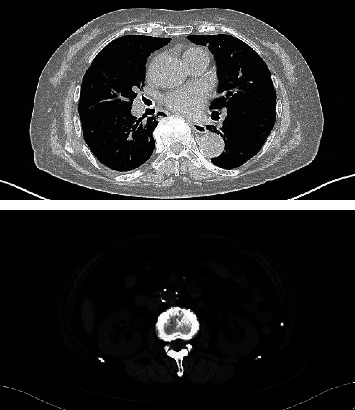
Plain CT of the chest and abdomen showing no findings suggestive of pneumonia, and no abnormality in renal morphology.

**Figure 2:**
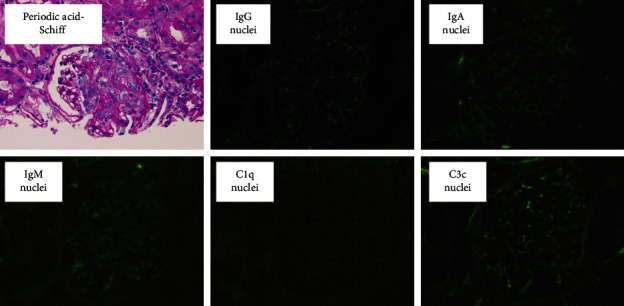
Histopathological findings in a kidney biopsy confirming pauci-immune crescentic glomerulonephritis. Periodic acid-Schiff stain showing a glomerulus with a cellular crescent arising in Bowman's space (original magnification, × 40). Immunofluorescence microscopy findings of a kidney biopsy. No significant deposits of immunoglobulins or complement components were found (original magnification, × 40).

**Figure 3:**
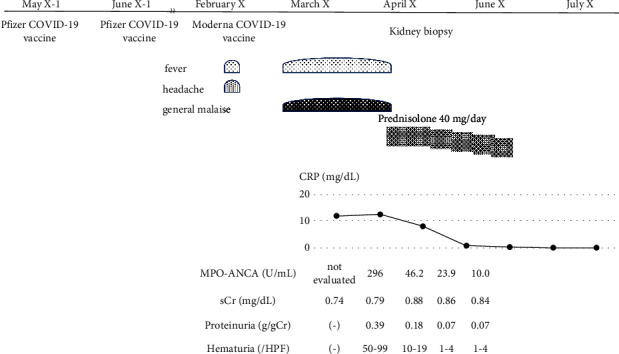
Clinical course.

A bacterial infection was suspected, and 2 g of ceftriaxone was administered daily for 7 days. However, the symptoms did not improve, and the patient was then admitted to our hospital. The physical examination and imaging tests showed no findings suggestive of pyrexia, and there were no abnormalities in renal morphology (Figure 1). Although pyuria was not observed, microscopic hematuria and urinary protein was found and MPO-ANCA was as high as 296 IU/mL. Thus, MPO-ANCA AAV was suspected (Table 1). Antinuclear antibody, proteinase 3-antineutrophil cytoplasmic antibody, and glomerular basement membrane antibody were negative. She had not taken drugs such as propylthiouracil, her blood cultures were negative, and there were no findings suggestive of infective endocarditis. Specimens obtained by renal biopsy are shown in Figure 2. Specimens had a cortex: medullary ratio of 9 : 1, and 26 glomeruli were observed. Cellular or fibrocystic crescent formation was observed in 6 glomeruli. No increase in mesangial matrix or cells was evident, and mild inflammatory cell infiltration was diffusely observed in the renal tubules and interstitium. Immunofluorescence confirmed pauci-immune glomerulonephritis. MPO-ANCA was positive and showed rapidly progressive glomerulonephritis, while polyarteritis nodosa and IgA vasculitis were negative. No pulmonary lesions or neurological lesions were observed, and based on pathological findings, she was diagnosed with renal-limited MPO-ANCA AAV. Steroids were started with prednisolone at 40 mg/day (0.8 mg/kg), and general symptoms such as pyrexia, malaise, and inflammatory reaction, as measured by the blood test, improved, and urinary occult blood and urinary protein disappeared. Steroids were tapered to 20 mg/day, and her condition remains stable (Figure 3).

## 3. Discussion

This case adds to previous evidence that MPO-ANCA AAV can occur after the COVID-19 vaccination.

In this study, we report a case of myeloperoxidase antineutrophil cytoplasmic antibody (MPO-ANCA)-associated vasculitis, which developed after a third booster dose of Moderna COVID-19 vaccination in an 82-year-old woman. Although she initially presented with persistent pyrexia and general malaise one month after vaccination, blood testing revealed a high level of MPO-ANCA and microscopic hematuria. This case illustrates that in patients developing pyrexia, prolonged general malaise, urinary occult blood, or renal impairment following Moderna COVID-19 vaccination, the onset of MPO-ANCA-associated vasculitis should be considered.

Although the patient had been under treatment for hypertension, neither renal impairment nor urinalysis abnormalities had been detected prior to the present condition. Further, blood and urine tests performed 1 month before the third COVID-19 booster vaccination showed no particular abnormalities. Millions of people are being vaccinated around the world [8], and it is conceivable that people may develop conditions temporally associated with vaccination but which are causally unrelated to the vaccine itself. Nevertheless, the fact that no laboratory abnormalities were found before the third booster vaccination in this patient suggests that COVID-19 vaccination was involved in the onset of MPO-ANCA AAV. ANCA AAV is known to occur with certain medications such as propylthiouracil [9], as well as infections, and malignant tumors [10]. Further, some reports have described the development of ANCA AAV after influenza vaccination [4, 5]. The relationship between influenza vaccines and the onset of ANCA AAV might be explained by the possibility that vaccination of individuals with a genetic predisposition may induce autoimmune phenomena [4]. Two possible mechanisms are assumed to explain vascular wall impairment in the context of autoimmunity caused by vaccines: an antigen-specific reaction caused by molecular homology between vascular wall components and bacterial cell components; and an antigen-nonspecific reaction caused by the vaccine that induces a bystander effect and activates autoreactive T cells in a nonspecific manner [5]. It has also been noted that autoantigens, which are not normally present, are also involved due to a vaccine-induced increase in cytokine production or abnormality in expression of MHC class II molecules [11]. mRNA vaccines may cause differential stimulation of myeloid and dendritic cells, which thereby activates the downstream pathway to produce autoinflammation [12]. mRNA vaccines have a lesser risk of infection and insertion-related mutagenesis but generate antiviral neutralizing immunoglobulins and stimulate strong immune responses by activating CD8 + and CD4 + T cells [12]. mRNA vaccines may cause enhanced stimulation of innate and acquired immunity compared to inactivated vaccines or natural infection [12, 13]. This new-onset autoinflammation occurs in genetically predisposed individuals, among whom those with compromised immune systems have decreased clearance of nucleic acids. This, in turn, predisposes them to Neutrophil Extracellular Traps (NETs) [14], which are highly proinflammatory and provide a sustained antigenic stimulus. This so-called NETosis is a critical step in the pathogenesis of both cytokine storms in COVID-19 infection [15] and COVID-19-triggered AAV [16]. Nevertheless, the exact mechanism is not fully understood. Accumulation of further cases will allow elucidation of the mechanism and causal relationship between COVID-19 vaccination and the onset of ANCA AAV.

Recently, a literature review of vaccine-associated AAV after COVID-19 vaccination was published [17]. According to the review, most cases were secondary to mRNA vaccines (75.8%), with 24 patients developing new onset AAV after vaccination (82.7%). Symptoms such as hematuria and renal failure often occurred within one month of vaccination. Regarding ANCA subtypes of AAV, 15 cases were positive for MPO, 4 for PR3, and 3 for both (MPO and PR3). Of the 29 cases reviewed, 27 had renal involvement (93.1%), and 10 had both lung and kidney involvement (34.4%). Most patients received immunosuppressive treatment, such as steroids, rituximab, or cyclophosphamide. Ten of these 27 patients improved (37.0%), 9 showed partial improvement, and 5 were dependent on dialysis. They all responded relatively well to immunosuppressive therapy. In this case, the symptoms appeared one month after the third vaccination, only MPO-ANCA was positive, there were no lung lesions but rather only renal failure, and the response to steroid treatment was good. These findings are consistent with existing reports. There is also a report of ANCA-associated vasculitis after two doses of the mRNA BNT162b2 COVID-19 vaccine (Pfizer) and a third dose of the mRNA-1273 COVID-19 vaccine (Moderna), similar to this case [18]. A recent comparative study on using the same vaccine as the primary series (homologous boosters) versus using a different vaccine (heterologous boosters) showed that the immunogenicity of heterologous boosting was generally similar to or greater than those of homogenous boosting [19]. Although this may have been involved in the emergence of renal injury after vaccination in our present case, further studies are required to confirm whether the increased immune response of heterologous injection of COVID-19 vaccinations contributes to a higher incidence of ANCA-associated vasculitis.

In addition to ANCA AAV, the following conditions have been reported to develop as glomerulonephritis after COVID-19 vaccination: IgA nephropathy, minimal change disease, antiglomerular basement membrane disease, and membranous nephropathy [20]. An observational study in Japan reported that macroscopic hematuria and proteinuria frequently occur after COVID-19 vaccination [21]. Accordingly, meticulous attention should be paid to urinary findings after COVID-19 vaccination.

The COVID-19 vaccination strategy is ongoing worldwide because mRNA vaccines are highly effective in preventing COVID-19 and reducing the seriousness of COVID-19 in the era of the SARS-CoV-2 (COVID-19) pandemic. Although common adverse reactions to COVID-19 vaccines include pyrexia and general malaise, MPO-ANCA AAV can occur after COVID-19 vaccination. If pyrexia, prolonged general malaise, urinary occult blood, or renal impairment is observed, the onset of MPO-ANCA AAV should be considered.

## Figures and Tables

**Table 1:** Laboratory data.

Parameter	Value
White blood cells (^*∗*^/*μ*L)	13800
Neutrophils (%)	86.8
Lymphocytes (%)	5.3
Monocytes (%)	2.9
Eosinophils (%)	4.1
Basophils (%)	0.9
Red blood cells (×10^4^/*μ*L)	361
Hemoglobin (g/dL)	10.4
Hematocrit (%)	32.1
Platelet count (×10^4^/*μ*L)	12.2
Total protein (g/dL)	5.8
Albumin (g/dL)	2.1
Total bilirubin (mg/dL)	0.4
Aspartate aminotransferase (U/L)	21
Alanine aminotransferase (U/L)	19
Lactate dehydrogenase (U/L)	175
*γ*-Glutamyl transpeptidase (U/L)	29
Creatine kinase (U/L)	24
Blood urea nitrogen (mg/dL)	11.7
Creatinine (mg/dL)	0.79
Sodium (mEq/L)	138
Potassium (mEq/L)	3.5
Chloride (mEq/L)	102
Hemoglobin A1c (%)	7.0
C-reactive protein (mg/dL)	12.1
C3 (mg/dL)	145
C4 (mg/dL)	27.7
Immunoglobulin G (mg/dL)	1775
Immunoglobulin A (mg/dL)	302
Immunoglobulin M (mg/dL)	100
Hepatitis B virus antigen	Negative
Hepatitis C virus antibody	Negative
Cryoglobulin	Negative
Antinuclear antibody	Negative
Myeloperoxidase-antineutrophil cytoplasmic antibody (U/mL)	296
PR3-ANCA:proteinase3-antineutrophil cytoplasmic antibody (U/mL)	Negative
Glomerular basement membrane antibody (U/mL)	Negative
SARS-CoV2-polymerase chain reaction	Negative

<Urinalysis>	
Protein	1+
Occult blood	2+
Glucose	—
Red blood cells (/HPF)	30–49
White blood cells (/HPF)	5–9
Protein excretion (g/gCr)	0.39
N-acetyl-*β*-D-glucosaminidase (U/L)	8.5
Β2-macroglobulin (*μ*g/L)	197

HPF, high-power field.

## Data Availability

The data supporting the findings of this study are included within the article.
